# Tunneled Tunica Vaginalis Flap for Recurrent Urethrocutaneous Fistulae

**DOI:** 10.1155/2008/615928

**Published:** 2008-11-04

**Authors:** Jonathan C. Routh, James J. Wolpert, Yuri Reinberg

**Affiliations:** ^1^Department of Urology, Mayo Clinic, Rochester, MN 55905, USA; ^2^Division of Pediatric Urology, Pediatric Surgical Associates, Minneapolis, MN 55404, USA

## Abstract

The tubularized incised plate (TIP) hypospadias repair is currently the most widely used urethroplasty technique. The most significant post-TIP complication is urethrocutaneous fistula (UCF) development. Tunneled tunica vaginalis flap (TVF) is a well-described technique for the repair of UCF. We retrospectively reviewed all patients undergoing repeat repair of UCF after TIP repair from 2001 to 2005. Twelve boys underwent TVF repair at our institution for recurrent UCF. Fistulae ranged from distal penile to penoscrotal in location. Median surgical time was 45 minutes and no postoperative complications occurred. After a median follow-up of 32 months (range 16–48 months), no patient has yet had a recurrence of UCF. In conclusion, TVF repair is a successful technique for the treatment of UCF after previous failed repair. TVF is technically simple to perform and should be considered for treating UCF following TIP urethroplasty, particularly in a repeat surgical setting.

## 1. INTRODUCTION

Hypospadias has been recognized as a surgically treatable condition for nearly two millennia. Physicians of both the Hellenic (Heliodorus and Antyllus) and Roman worlds (Celsus and Galen) described the condition as well as its possible surgical remedies [1, 2]. Needless to say, surgical techniques have evolved with time. The most significant recent advance in this evolution occurred in 1994 with Snodgrass' description of the tubularized incised plate (TIP) urethroplasty technique [3]. Since its initial description in cases of distal hypospadias, TIP urethroplasty has now been applied with notable success to both proximal [4] and reoperative [5, 6] hypospadias.

Despite these advances in urethroplasty technique, certain complications remain problematic for the modern hypospadiologist, namely, meatal stenosis, urethral stricture or diverticulum, wound dehiscence, and, perhaps most importantly, urethrocutaneous fistulae (UCF). A recent review of TIP urethroplasty reveals a combined UCF rate of 2.4% across several major centers [7]. The interposition of vascularized tissue such as tunica vaginalis flap (TVF) or deepithelialized dartos tissue has been suggested as an effective means of reducing UCF formation rate in multiple urethroplasty techniques, including TIP [7–9].

Successful fistula repair depends in large part upon meticulous attention to surgical detail, as well as the use of interposed tissue. In reoperative patients, however, this can be difficult to achieve, as well-vascularized tissues amenable to interposition may be sparse. One possibility for interposition graft for UCF repair is the use of tunneled TVF, which was first described in 1970 by Hosli [10] and subsequently popularized by Snow et al. [8, 9]. Despite the widespread use of both TIP urethroplasty and UCF repair, there is a lack of data describing TVF use for UCF following TIP urethroplasty. We have previously examined our results with the use of TVF for UCF following TIP urethroplasty [11]. We now update that experience in the most difficult patients, those who have undergone multiple previous penile surgeries including previous failed UCF repair following TIP.

## 2. MATERIALS AND METHODS

### 2.1. Patient selection

We retrospectively reviewed all records of patients undergoing TVF repair of post-TIP UCF at our practice between January 2000 and December 2005. Only patients who had failed previous UCF repair were included in this study. All final UCF repairs were performed by the authors, although initial urethroplasty was performed elsewhere in some patients. Surgeries were performed under general anesthesia in our outpatient surgical facility with a 6-month minimum healing period between the previous penile surgery and UCF repair. For each patient, we abstracted the following data: age, surgeon, number of previous surgeries, number and location of fistulae, original pre- and postoperative location of urethral meatus, surgical/anesthetic duration, length of followup, and postoperative complications. We loosely defined complications to include meatal stenosis, postoperative wound infection, scrotal hematoma, penile torque, penile tethering, or recurrence of fistulae.

### 2.2. Surgical technique

Our surgical technique has been previously described [11]. Briefly, all patients undergo calibration of the distal urethra. Dilute Betadine solution is then instilled into the urethra through the meatus to visually confirm the exact location and number of all UCF. A Foley catheter is placed in the bladder. Each UCF tract is excised, and the urethra is primarily closed in two layers using 7-0 PDS suture. A 1-cm incision is then made at the penoscrotal junction and a flap of tunica vaginalis is harvested, taking great care to avoid inclusion of cremasteric muscle fibers. Flap length is determined by the distance from the harvest site to the UCF site. The TVF is then tunneled underneath the penile skin and brought out through the most distal UCF tract. The TVF is then fixed at each UCF site using 7-0 PDS. The skin overlying each UCF tract is then closed.

## 3. RESULTS

Twelve boys (mean age 2.2 years) underwent TVF repair of recurrent UCF at our institution during the study period. Six boys had distal shaft and 6 had proximal shaft or penoscrotal hypospadias. All patients had originally undergone TIP repair with subsequent UCF formation; 4 patients (33%) had their initial urethroplasty performed at our institution, with the remainder being referred after initial TIP repair done elsewhere. Eleven fistulae (92%) developed spontaneously within the first 2 years after initial TIP repair. One patient did well postoperatively but ultimately developed a UCF after direct trauma to the penis.

All boys had undergone previous attempted UCF repair, with 8 (67%) undergoing 1 repair, 2 (17%) undergoing 2 repairs, and 1 each (8%) undergoing 3 and 4 previous repairs. All patients had a subcoronal UCF, while 8 (67%) were found to have multiple fistulae along the distal shaft: 4 each (33%) with 3 and 4 fistulae, including 1 patient with a penoscrotal fistula. Median operative time was 45 minutes (range 30–90).

No pre- or postoperative complications have as yet occurred in any patient, including hematoma, wound infection, abscess, or secondary chordee or torque. After a mean followup of 32 months (range 16–48 months), no patient has yet had a recurrence of their UCF.

Following our initial experience with the patients described above, we have since performed this procedure on an additional 10 children, none of whom have had a fistula recurrence in followup of less than one year.

## 4. DISCUSSION

The use of TIP urethroplasty has greatly increased since its initial introduction by Snodgrass in 1994 [3], with several centers reporting it to, now, be their primary urethroplasty technique [5, 12]. However, the procedure is not without potential complications, including meatal stenosis, urethral stricture or diverticulum, wound dehiscence, and UCF. In a recent review, a UCF rate of 2.4% was noted [7]. Several reports, however, show a much higher fistula rate, including those of Chatterjee et al. (15%) [13], Amukele et al. (17%) [14], and Guralnick et al. (16%) [15].

Snow et al. have advocated the preventative use of TVF during primary hypospadias repair. When combined with use of the operative microscope, their reported posturethroplasty UCF rate is 0%, with a 2.2% complication rate, namely, scrotal hematoma and abscess [8]. The utilization of TVF has also been described as a means of UCF repair following initial urethroplasty using several techniques, with a combined recurrence rate of 7.9% and no complications reported [16–20]. Pattaras and Rushton have reported two patients who developed severe penile torque several years after primary urethroplasty using TVF in a preventative manner. In both cases the flap was simply divided with subsequent resolution of the torque [21].

The advantages of TVF are myriad, particularly in the reoperative patient. As the tunica lies well away from the operative field of the penile shaft, its blood supply remains uninterrupted even in the setting of numerous reoperations. Operative access to tunica vaginalis is technically simple, as evidenced by our median anesthesia time of 45 minutes. The low complication rate in our hands, which is consistent with previous reports [8, 16–18, 20, 21], provides further evidence that the procedure is safe and easy to perform, even in the setting of a reoperative patient with multiple fistulae. Obviously, strict adherence to the basic principles of UCF and hypospadias repair should be maintained.

The limitations of this study are primarily a question of length of followup; as noted by Pattaras and Rushton [21], several years may need to elapse before the development of truly long-term complications. Our relatively small sample size and average followup of 32 months may not be adequate to fully demonstrate all complications and thus may falsely lower our complication rate. However, it should be noted that complication rates are low even in studies with significantly longer followup [8]. This lends a measure of reassurance that our rates are not overly optimistic. Further, we feel that many complications can be successfully avoided by careful attention to detail—specifically, ensuring that the TVF is of adequate length in order to avoid secondary chordee and that no cremasteric fibers are included with the flap in order to avoid penile torque. By following these simple principles, a technically simple, highly successful repair of recurrent UCF can be accomplished in minimal operative time.

## 5. CONCLUSIONS

Tunneled TVF repair is a highly successful technique for the treatment of posturethroplasty recurrent UCF. The technique is simple and quick to perform with no complications encountered in our experience. TVF repair is particularly useful as the tissue of choice for treating UCF in the repeat surgical setting following initial failed TIP urethroplasty, with excellent results at long-term followup.

## References

[1] Lascaratos J, Kostakopoulos A, Louras G (1999). Penile surgical techniques described by Oribasius (4th century CE). *BJU International*.

[2] Smith ED (1997). The history of hypospadias. *Pediatric Surgery International*.

[3] Snodgrass W (1994). Tubularized, incised plate urethroplasty for distal hypospadias. *The Journal of Urology*.

[4] Snodgrass W, Koyle M, Manzoni G, Hurwitz R, Caldamone A, Ehrlich R (1998). Tubularized incised plate hypospadias repair for proximal hypospadias. *The Journal of Urology*.

[5] Borer JG, Bauer SB, Peters CA (2001). Tubularized incised plate urethroplasty: expanded use in primary and repeat surgery for hypospadias. *The Journal of Urology*.

[6] Snodgrass WT, Lorenzo A (2002). Tubularized incised-plate urethroplasty for hypospadias reoperation. *BJU International*.

[7] Snodgrass WT (1999). Tubularized incised plate hypospadias repair: indications, technique, and complications. *Urology*.

[8] Snow BW, Cartwright PC, Unger K (1995). Tunica vaginalis blanket wrap to prevent urethrocutaneous fistula: an 8-year experience. *The Journal of Urology*.

[9] Snow BW (1986). Use of tunica vaginalis to prevent fistulas in hypospadias surgery. *The Journal of Urology*.

[10] Hosli PO (1970). Eine technik zum verschluss von harnrohrenfistein. *Urologe*.

[11] Routh JC, Wolpert JJ, Reinberg Y (2006). Tunneled tunica vaginalis flap is an effective technique for recurrent urethrocutaneous fistulas following tubularized incised plate urethroplasty. *The Journal of Urology*.

[12] Steckler RE, Zaontz MR (1997). Stent-free Thiersch-Duplay hypospadias repair with the Snodgrass modification. *The Journal of Urology*.

[13] Chatterjee US, Mandal MK, Basu S, Das R, Majhi T (2004). Comparative study of dartos fascia and tunica vaginalis pedicle wrap for the tubularized incised plate in primary hypospadias repair. *BJU International*.

[14] Amukele SA, Stock JA, Hanna MK (2005). Management and outcome of complex hypospadias repairs. *The Journal of Urology*.

[15] Guralnick ML, al-Shammari A, Williot PE, Leonard MP (2000). Outcome of hypospadias repair using the tubularized, incised plate urethroplasty. *The Canadian Journal of Urology*.

[16] Landau EH, Gofrit ON, Meretyk S, Katz G, Golijanin D, Shenfeld OZ, Pode D (2003). Outcome analysis of tunica vaginalis flap for the correction of recurrent urethrocutaneous fistula in children. *The Journal of Urology*.

[17] Kirkali Z (1990). Tunica vaginalis: an aid in hypospadias surgery. *British Journal of Urology*.

[18] Singh RB, Pavithran NM (2004). Tunica vaginalis interposition flap in the closure of massive disruption of the neourethral tube (macro urethrocutaneous fistulae). *Pediatric Surgery International*.

[19] Shankar KR, Losty PD, Hopper M, Wong L, Rickwood AMK (2002). Outcome of hypospadias fistula repair. *BJU International*.

[20] Voges GE, Riedmiller H, Hohenfellner R (1990). Tunica vaginalis free grafts for closure of urethrocutaneous fistulas. *Urologia Internationalis*.

[21] Pattaras JG, Rushton HG (1999). Penile torque after the use of tunica vaginalis blanket wrap as an aid in hypospadias repair. *The Journal of Urology*.

